# Supplementary material for: Prognostic Value of the Controlling Nutritional Status (CONUT) Score in Patients at Dialysis Initiation
Source: Nutrients. 2022 May 31;14(11):2317. doi: 10.3390/nu14112317 (PMC9182995; doi:10.3390/nu14112317)
Supplement: Supplementary file 1 [file nutrients-14-02317-s001.zip › nutrients-1750809-supplementary.pdf]

Table S1. Nutritional status by CONUT score.

Parameter	Normal	Light	Moderate	Severe
Albumin, g/dL	≥ 3.50	3.00–3.49	2.50–2.99	< 2.50
Score	0	2	4	6
Total lymphocyte count, /μL	≥ 1600	1200–1599	800–1199	< 800
Score	0	1	2	3
Total cholesterol, mg/dL	≥ 180	140–179	100–139	< 100
Score	0	1	2	3
Total score	0–1	2–4	5-8	9–12

Abbreviation: CONUT, controlling nutritional status

Table S2. The univariate Cox proportional hazards analysis of predictive value for all-cause mortality, CVDs mortality, and infectious diseases mortality.

Variable	All-cause mortality			Infectious diseases mortality			CVDs mortality		
	HR	95% CI	P value	HR	95% CI	P value	HR	95% CI	P value
CONUT score	1.18	1.09–1.27	**<0.0001**	1.3	1.14–1.48	**<0.0001**	1.12	0.99–1.27	0.0593
Age	1.05	1.03–1.07	**<0.0001**	1.05	1.02–1.09	**0.0036**	1.05	1.02–1.08	**0.0038**
Sex, male	1.4	0.88–2.23	0.1568	1.23	0.55–2.75	0.6085	2.87	1.12–7.35	**0.028**
Smoking	0.98	0.66–1.46	0.9337	0.99	0.5–2.0	0.9939	0.99	0.52–1.85	0.9644
History of CVD	2.15	1.44–3.21	**0.0002**	1.53	0.76–3.08	0.2292	3.93	1.96–7.88	**0.0001**
Late referral	1.16	0.63–2.12	0.6362	1.23	0.43–3.5	0.7008	1.51	0.63–3.61	0.3524
Diabetes mellitus	0.69	0.47–1.03	0.0679	0.83	0.41–1.66	0.5922	0.69	0.37–1.29	0.2424
Hypertension	0.79	0.44–1.38	0.4033	1.17	0.36–3.86	0.7931	0.7	0.29–1.68	0.4242
Dyslipidemia	0.58	0.37–0.91	**0.0181**	0.65	0.29–1.4	0.2699	0.5	0.24–1.06	0.0704
BMI	0.93	0.88–0.99	**0.0196**	0.91	0.82–1.01	0.0709	0.97	0.89–1.06	0.4668
CTR	1.01	0.98–1.03	0.6144	1.02	0.98–1.07	0.3	0.99	0.96–1.04	0.9222
Dialysis modality, PD	0.76	0.42–1.39	0.3777	0.58	0.18–1.9	0.3685	0.64	0.23–1.8	0.4004
CRP (mg/dL)	1.23	1.13–1.34	**<0.0001**	1.18	0.99–1.41	0.0619	1.24	1.12–1.37	**<0.0001**
Hemoglobin (g/dL)	0.88	0.77–1.01	0.0675	0.84	0.67–1.07	0.1569	1.01	0.81–1.25	0.9222

Abbreviations: CVD, cardiovascular disease; HR, hazard ratio; CI, confidence interval; CONUT, controlling nutritional status; BMI, body mass index; CTR, cardi thoracic ratio; PD, peritoneal dialysis; CRP, C–reactive protein.
